# Hepatic Radioembolization as a Bridge to Liver Surgery

**DOI:** 10.3389/fonc.2014.00199

**Published:** 2014-07-30

**Authors:** Arthur J. A. T. Braat, Julia E. Huijbregts, I. Quintus Molenaar, Inne H. M. Borel Rinkes, Maurice A. A. J. van den Bosch, Marnix G. E. H. Lam

**Affiliations:** ^1^Department of Radiology and Nuclear Medicine, University Medical Center Utrecht, Utrecht, Netherlands; ^2^Department of Surgery, University Medical Center Utrecht, Utrecht, Netherlands

**Keywords:** radioembolization, liver malignancies, downstaging, bridge to transplantation, transplantation, future liver remnant, hepatobiliary scintigraphy, dosimetry

## Abstract

Treatment of oncologic disease has improved significantly in the last decades and in the future a vast majority of cancer types will continue to increase worldwide. As a result, many patients are confronted with primary liver cancers or metastatic liver disease. Surgery in liver malignancies has steeply improved and curative resections are applicable in wider settings, leading to a prolonged survival. Simultaneously, radiofrequency ablation (RFA) and liver transplantation (LTx) have been applied more commonly in oncologic settings with improving results. To minimize adverse events in treatments of liver malignancies, locoregional minimal invasive treatments have made their appearance in this field, in which radioembolization (RE) has shown promising results in recent years with few adverse events and high response rates. We discuss several other applications of RE for oncologic patients, other than its use in the palliative setting, whether or not combined with other treatments. This review is focused on the role of RE in acquiring patient eligibility for radical treatments, like surgery, RFA, and LTx. Inducing significant tumor reduction can downstage patients for resection or, through attaining stable disease, patients can stay on the LTx waiting list. Hereby, RE could make a difference between curative of palliative intent in oncologic patient management. Prior to surgery, the future remnant liver volume might be inadequate in some patients. In these patients, forming an adequate liver reserve through RE leads to prolonged survival without risking post-operative liver failure and minimizing tumor progression while inducing hypertrophy. In order to optimize results, developments in procedures surrounding RE are equally important. Predicting the remaining liver function after radical treatment and finding the right balance between maximum tumor irradiation and minimizing the chance of inducing radiation-related complications are still challenges.

## Introduction

According to recent estimations, an increase in the global cancer burden is expected from 12.7 million new cases in 2008 to 22.2 million by 2030 (1). As these numbers grow, so does the number of patients with liver malignancies. As primary liver cancer, hepatocellular carcinoma (HCC) is the fifth most common cancer worldwide. In metastatic liver disease, the incidence of colorectal carcinoma (CRC) is high. It is the third most common cancer worldwide (2). At the time of diagnosis, 14.5% of the patients present with synchronous metastatic liver disease, of which 76.8% is limited to the liver. Another 12.8% develops metachronous liver metastases within 5 years after initial diagnosis (3). Many other tumor cell types, including neuroendocrine tumors (NET), cholangiocarcinoma (CC), and others, frequently present as liver-dominant disease (4). By the time that the disease has spread to the liver it is often difficult to treat with low response rate and a dismal survival. For example, in patients with CRC presenting with synchronous liver metastases, without palliative chemotherapy survival is only 5–7 months. With palliative chemotherapy survival increases to 22 months (5–7). In patients with unifocal HCC (<5 cm) a 5-year survival of 7% and a median survival of 18 months is seen without resection. After surgical resection this increases to a 33% and 47 months, respectively (8).

These numbers indicate a clear need for improvement of current treatment strategies for liver malignancies. In recent years, trans-arterial yttrium-90 (^90^Y) radioembolization (RE) has gained rapid interest in the management of liver malignancies. High response rates and a favorable toxicity profile make this an elegant therapy, even in patients with underlying cirrhotic liver disease. Compared to trans-arterial chemo-embolization (TACE), which is commonly used in patients with HCC, RE results in similar response rates, a comparable overall survival (OS) and less adverse events (9–12). Currently, many phase 2–3 clinical studies on RE are recruiting patients for efficacy evaluation and toxicity screening in patients with primary liver malignancies compared to current treatments [YES-P (13), SARAH (14), and SIRVENIB (15)] or combined with current treatments [SORAMIC (16) and STOP-HCC (17)]. In secondary liver malignancies, RE is investigated in phase 2–3 trials for efficacy evaluation and toxicity combined with chemotherapeutic regimes. Simultaneously, determining the place of RE in the treatment algorithm as a first-line, second-line, or salvage therapy [SIRFLOX (18), EPOCH (19), FOXFIRE, and SIR-step (20)]. At this point, RE is mostly used as an end of line treatment modality. However, RE can also be applied in a pre-operative setting before hepatic surgery, before ablative treatments such as radiofrequency ablation (RFA), or before a combination of those. In this review, the possible merits of RE in the pre-surgical setting will be discussed.

Surgical eligibility depends on many factors, currently rendering 10–30% of HCC patients and 5–9% of CRC patients eligible for primary surgical resection (5, 6, 21–23). When deemed ineligible for radical treatments, providing sufficient tumor reduction, called downstaging, might allow for radical treatments. Once downstaging has occurred, selected patients could be eligible for liver transplantation (LTx). However, availability of liver donors is limited and minimizing the chance of tumor progression while on the waiting list is needed, since disease progression and death occur in 10–23% of the patients while being listed (24, 25). Prevention of disease progression during this waiting period is called a bridge to transplantation, and will be discussed in our second section. Once patients are eligible for resection (not for transplantation), a sufficient post-operative liver reserve is needed to avoid post-operative complications and death due to hepatic failure. Inducing hypertrophy of the future remnant liver (FRL) by portal vein embolization (PVE) is an accepted method to minimize the chance of post-operative hepatic failure. After PVE, it takes about 3–6 weeks to induce adequate hypertrophy and around 17.5% of the patients experience tumor progression during this time interval, making them ineligible for resection (26). In unilobar RE, hypertrophy of the non-treated lobe has been described. Maybe using RE instead of PVE to achieve hypertrophy could help overcome this problem of tumor progression.

Using RE in the pre-operative setting, when patients may still be treated with curative intent, requires special attention to issues of efficacy and toxicity. After a short introduction to RE treatment itself, the role of RE in downstaging disease and as a bridge to LTx will be discussed. The role of RE in optimizing the future liver remnant will also be discussed, as well as issues of dosimetry and treatment accuracy.

## Radioembolization

Radioembolization is a liver-directed treatment using radioactive microspheres. It is based on the dual blood supply of the liver (i.e., the portal vein and the hepatic artery). The contribution of the portal vein and hepatic artery to the blood flow of the normal liver parenchyma is circa 70 and 30%, respectively (27). For liver malignancies, the hepatic artery is the primarily blood supply (28). RE uses these perfusion differences between tumors and non-tumorous tissue to its advantage. The administered microspheres mainly lodge in the tumor arterioles, leading to a high tumor absorbed dose and a limited absorbed dose to the healthy liver parenchyma. This is even more the case for hypervascular tumors such as HCC and NET. In RE treatment planning these and several other factors play an important role.

Adequate patient selection is required first of all. Performance score (ECOG 0–2), adequate liver function (Child Pugh score A or B; bilirubin levels <2 mg/dl), liver-dominant, or liver-limited disease and a life expectancy of >3 months are of particular importance (22, 29). When patients comply with these initial criteria, angiography follows. Injection positions for RE are planned during angiography, necessary precautions are taken to prevent extrahepatic deposition of microspheres, and the distribution of the microspheres is simulated by injecting ^99m^Tc-macroaggregated albumin (^99m^Tc-MAA). This is followed by planar scintigraphy and SPECT(/CT) imaging to detect possible extrahepatic deposition in abdominal organs and the lungs.

Once extrahepatic depositions have been excluded, treatment activity may be calculated. Different methods apply for the different microspheres commercially available. Resin microspheres (SIR-Spheres^®^, SIRTeX Medical Limited, Lane Cove, NSW, Australia) and glass microspheres (TheraSphere^®^, BTG International Ltd., Canada) are both FDA approved. Both have different activity calculation methods defined by the manufacturer (Table 1). One method is advocated for glass microspheres, while three methods can be used for resin microspheres; the so-called empirical, body surface area (BSA) and partition method (Table 1).

**Table 1:** **Dose calculation methods**.

Microspheres		Dose calculation method
SIR-spheres user manual (30)	Empirical	Tumor load ≤25% = 2 GBq whole-liver delivery
		Tumor load 25–50% = 2,5 GBq whole-liver delivery
		Tumor load ≥50% = 3 GBq whole-liver delivery
	Body surface area (BSA)	$\text{A}\left(\text{GBq}\right)=\left(\text{BSA}-0.2\right)+\left[\frac{\text{tumor}\;\text{volume}}{\text{tumor volume + liver volume}}\right]$ in which:
		BSA = 0.20247 x height(m)^0.725^ × weight(kg)^0.425^
	Segmentation	$\text{A}\left(\text{GBq}\right)=\frac{\text{D}\left(\text{Gy}\right)\times \left(\left[\text{T}∕\text{N}\times {\text{Mass}}_{\text{tumor}}\right]+{\text{Mass}}_{\text{liver}}\right)}{49670\;\times \;\left(1-\frac{\text{lung shunt fraction}\left(\%\right)}{100}\right)}$ in which:
		$\text{T}∕\text{N}=\frac{\text{Activit}{\text{y}}_{\text{tumor}}∕\text{Mas}{\text{s}}_{\text{tumor}}}{\text{Activit}{\text{y}}_{\text{liver}}∕\text{Mas}{\text{s}}_{\text{liver}}}$ based on MAA-SPECT/CT
TheraSpheres user manual (31)		$\text{A}\left(\text{GBq}\right)=\frac{\text{D}\left(\text{Gy}\right)\times \text{Mas}{\text{s}}_{\text{liver}}\left(\text{kg}\right)}{50\times \left(1-\frac{\text{Lung shunt fraction(}\%\text{)}}{100}\right)}$ with an upper limit of lung shunt dose: lung shunt fraction(%) × A(GBq) = 0.61 GBq

*A, desired activity of microspheres; D, nominal dose to the liver; T/N, tumor to non-tumor ratio*.

After RE treatment, ^90^Y-brehmsstralung SPECT or ^90^Y-PET is conducted to evaluate the distribution of the microspheres, excluding extrahepatic depositions. Post-treatment imaging can also be used for dosimetry. RE is generally a safe therapy, with relatively few side effects. Most patients will experience a limited degree of acute side effects (<30 days after RE) at a constitutional (fatigue and fever), gastrointestinal (nausea, emesis, abdominal pain, and ulcer), or hepatic level (biochemically). Some might develop late radiation effects, like RE-induced liver disease (REILD), which may occur in up to 5% of patients treated with RE (29, 32, 33).

## Downstaging

Undoubtedly, surgery with curative intent is the most effective treatment strategy for a patient with liver malignancy. Literature has shown improved survival in HCC and CRC (liver metastases) after resection of all liver tumors (34, 35). Surgical eligibility depends on many factors, currently rendering 10–30% of the HCC patients and 5–9% of the CRC patients eligible for primary metastasectomy (5, 6, 21–23). Several contra-indications for surgical resection are in order: multiple bilobar tumors, inadvertent tumor localizations (near proximity to large blood vessels), inability to create sufficient resection margins (>10 mm), or an inadequate residual liver volume (liver remnant) (36, 37). Due to improvements in surgical techniques, the number of liver metastases in CRC has become less important and does not influence prognosis (36–38). Patients with ≥4 liver metastases in CRC show a significantly poorer survival after resection with a 5-year survival of 23% compared to patients with 1–3 liver metastasis with a 5-year-survival of 44% (39). In HCC, there is a direct relation between the amount of tumors and survival. In solitary HCC, a 5-year survival of 56% is seen and in all patients with multiple HCC’s survival is shorter than 3 years after resection (40–42).

As an alternative, RFA is a well-accepted treatment modality with good response rates in primary and secondary liver malignancies (43). Like surgical resection, near proximity to large vessels (“heat sink” effect; incomplete ablation) poses a problem (44, 45). Furthermore, the tumor must be reachable with the RFA-probe. RFA is adequate for tumors smaller than 3 cm to obtain complete necrosis, so tumor size should not exceed 3 cm (46). In a meta-analysis for HCC, surgical resection as primary treatment is superior to RFA with regard to recurrence rates, but surgical resection has more complications (47). For CRC results for RFA are similar to surgical resection (48).

In patients originally ineligible for resection/RFA, will downstaging followed by radical treatment [resection, RFA, and orthotopic liver transplantation (OLT)] truly lead to survival prolongation? A recent article by Ramanathan et al., described a 14-year experience of multiple treatments for HCC’s (25). Their population was analyzed retrospectively and divided in three groups. The first two groups were treated with an intention to transplantation down the road (goal: downstaging). The first group underwent transplantation eventually (Group 1, *n* = 139) and the second group did not receive transplantation, due to progressive disease (PD) (Group 2, *n* = 93). The third group had contra-indications for transplantation (Group 3, *n* = 484). Used treatments included TACE, trans-arterial chemoinfusion (TACI), RFA, resection, sorafenib, RE, or a combination. RE was not frequently used and rarely as downstaging modality in the first two groups (Group 1: 0/139, Group 2: 6/93, and Group 3: 55/484). The 5-year survival in the third group was only 4.4%. The second group showed a 5-year-survival of 35%, which was significantly worse than the transplantation-group, with a 5-year survival of 72.5% (25). This puts the need for downstaging in perspective. Once significant tumor reduction has occurred, patients with HCC can be treated with radical treatments leading to prolonged survival.

In patients with larger tumors (i.e., >3 cm; ineligible for RFA) or with tumors ineligible for resection, downstaging might be achieved by chemotherapy or biologicals, such as tyro-kinase inhibitors. These systemic agents, however, are commonly accompanied by (serious) adverse events. In order to gain a controlled and local tumor reduction, downstaging with RE seems a logical sequel. In contrast to surgery and RFA, tumor size and tumor localization pose less of a problem for RE. The role of RE for downstaging has predominantly been described in patients with HCC. No randomized controlled trials have been performed on downstaging patients using RE. Nonetheless, approximately 50% (range 29–67%) of the patients with HCC will be downstaged successfully (Table 2) (11, 49–52). Successful downstaging led to either resection, RFA, or OLT in three studies, in which approximately 1/3 of the downstaged patients were transplanted (10–23% of the total population) and 2/3 underwent resection or RFA (19–42% of the total population) (11, 49, 50). Two other studies focused on downstaging followed by OLT. Ibrahim et al. described eight patients with a caudate lobe HCC treated with RE. Four patients were downstaged successfully (50%) and three of them received OLT (37% of the total population) (51). Vouche et al. treated 102 patients ineligible for RFA or resection with RE, which led to OLT in 33 patients (32%); however, downstaging success rate was not described (53). The remaining study by Tohme et al. had a different study design, in which they retrospectively reviewed 20 transplanted patients that received RE as sole treatment as a bridge-to-transplant. Of these patients, 33% was downstaged according to imaging (52).

**Table 2:** **Response assessment and downstaging in HCC patients**.

Reference	*N*	mRECIST %		WHO %		EASL %		Downstaging success rate	Median time to response/downstaging^a^	Resection or RFA	OLT
		CR	PR	CR	PR	CR	PR	%	Months (range)	%^b^	%^b^
Kulik et al. (49)	34	NA	NA	NA	50	NA	NA	67	4 (1.9–16.3)	34	23
Lewandowski et al. (11)	43	NA	NA	0	61	47	39	58	3.1 (1.8–8.7)	42	21
Ibrahim et al. (51)	8	NA	NA	13	63	37	50	50	NA	NA	37
Iñarrairaegui et al. (50)	21	NA	NA	NA	NA	NA	NA	29	NA	19	10
Tohme et al. (52)	20	37	19	NA	NA	NA	NA	33	NA	NA	100^c^
Vouche et al. (53)	102	47	39	NA	NA	NA	NA	NA	NA	NA	32

**N*, number of patients, NA, data not available*.

*^a^In both studies defined as WHO PR*.

*^b^Percentage of total population*.

*^c^All patients received a liver transplantation (study design)*.

Out of six studies on downstaging prior to radical treatment, two studies described a median time to response, defined as partial response (PR) according to the World Health Organization (WHO) response criteria, of 3.1–4.2 months, significantly shorter than TACE with a median time to response of 10.9 months (11, 49). In concordance with literature on RE in a palliative setting, Table 2 shows a high PR rate and even complete response (CR) rate for HCC treated with RE. According to WHO, CR and PR was seen in 0–13 and 50–61%, respectively (11, 49, 51). By the European Association for the Study of the Liver criteria (EASL), CR and PR were even better 37–47 and 39–50%, respectively (11, 51). The most recent articles have implemented modified response evaluation criteria in solid tumors (mRECIST), which looks at tumor size as well as enhancing patterns. With mRECIST good results were shown with a CR and PR of 37–47 and 19–39%, respectively (52, 53). These numbers are also comparable to the numbers shown in an earlier published meta-analysis by Vente et al. (54).

In HCC, downstaging with RE seems feasible. Moreover, comparable response rates have been described for other liver malignancies. In metastatic CRC reported response rates ranged from 18 to 46%, in metastatic NET around 63% and in metastatic breast carcinoma they ranged from 26 to 91% (55–57). Like RE in HCC patients, randomized controlled trials are needed to better define the role of RE in downstaging patients with primary or secondary liver malignancies.

As discussed above, high response rates can be observed after RE in many different tumor types. RE as mono-therapy can induce CR, for example in up to 47% of HCC (see Table 2). The question is, how accurate are the current imaging modalities and its response criteria after RE therapy? Data of explanted livers show interesting results after RE in patients with HCC, who were downstaged for transplantation with RE. A correlation between radiologic response on follow-up imaging and the degree of necrosis found in the explanted specimen has been suggested (Table 3) (52, 58). In the study of Riaz et al., CR by EASL and PR by WHO correlated well with complete necrosis in their population of 33 transplantations, 1 surgical resection, and 1 autopsy. No enhancement of the lesions on imaging corresponded with complete necrosis in these cases. When using response criteria EASL had a 100% positive predictive value (PPV) and specificity, whereas WHO PR had a PVV and specificity of 78 and 71%, respectively (58). On the other hand, in an earlier study by Kulik et al. no correlation was described with WHO criteria. It was incorrect in five of the six explanted specimens and correct in the seventh resection specimen. However, all incorrect interpreted lesions (5/7) showed contrast enhancement on imaging (49). More recently, mRECIST has been introduced in HCC instead of WHO and EASL. Tohme et al. showed that four of the five patients (80%) with CR by mRECIST had complete necrosis in their explanted livers (52). In contrast, the more recent study by Vouche et al. found that only 7 of 14 patients (50%) with CR by mRECIST had complete necrosis at pathology (53).

**Table 3:** **Explanted data of HCC patients**.

Reference	Number of OLT	Degree of necrosis %			Comments on correlation imaging
	(% total population)				and histopathology
		100%	50–99%	0–50%	
Kulik et al. (49)	7 (24)	71	NA	NA	No correlation between imaging and histopathology
Riaz et al. (58)	35 (100) based on 38 lesions	61	24	15	EASL CR and WHO PR correlated well with complete necrosis
Ibrahim et al. (51)	3 (37)	33	66	0	
Tohme et al. (52)	20 (100)	25	30	45	Four of five patients with 100% necrosis had CR according to mRECIST
Vouche et al. (53)	33 (32)	52	48	0	Limitation of mRECIST; in CR 50% only partial necrosis

*NA, data not available*.

These discordant results highlight the limitations of current response criteria and its inability to consistently predict the degree of necrosis in treated liver malignancies. There is no primary role for ^18^F-fluorodeoxyglucose positron emission tomography (FDG-PET) in HCC, due to its low sensitivity of 50–55% in an overall HCC population (59, 60). Well-differentiated HCC’s show no to minimal FDG-uptake, while high to intense FDG-uptake can be seen in poorly differentiated/aggressive HCC’s (59–61). With this knowledge, individual prognostication seems possible (62). In the case of HCC and RE, currently two studies investigated the value of FDG-PET. Sabet et al. performed an FDG-PET before and after whole-liver RE in 33 patients. OS was best in FDG-negative HCC’s (13 months), followed by FDG-positive HCC’s that showed a metabolic response (defined as a SUV_max_ decrease of at least 20%; 10 months). Patients with FDG-positive HCC’s without metabolic response had the worst prognosis (OS 5 months) (63). Kucuk et al. investigated pre-treatment FDG-PET as a prognostication method in 19 patients. A longer progression free survival (PFS) was seen in the group with evident FDG-positive HCC’s prior to RE, compared to low FDG-positive or FDG-negative HCC’s prior to RE (20, 12, and 5 months, respectively). The author stated that the evident FDG-positive HCC’s responded better to RE (64).

^18^F-fluorodeoxyglucose positron emission tomography could be of better value to predict response in mCRC or metastases from other primary cancers than the conventional response criteria (65). A correlation between CEA and FDG-PET/CT has been described in a few studies (66–68). The metabolic response observed by PET-CT is based on a reduction in tumor load, and therefore, a decline in CEA. This does not always correlate with the response assessed on anatomical imaging (66, 68). Zerizer evaluated 25 patients with metastatic colon cancer to the liver with CECT and PET/CT. PFS at 2 years and decline in tumor markers were the primary end-points. Response on PET/CT was highly correlated with tumor markers (*p* < 0.0001) and prediction of PFS, while response on CT was not significantly correlated (68).

More PET/CT studies with histopathological correlation or correlation with patient outcome are needed. Hypothetically, if non-invasive imaging and its response criteria could better predict the degree of necrosis, patients could be stratified in time frames, giving clinicians better means to triage patients eligible for OLT.

## Bridge to Transplantation

Initially, the long-term results of OLT for HCC were disappointing with high recurrence rates and low survival. Early 30-day mortality was 21.3% after transplantation with septicemia and graft failure as leading causes and 5-year survival was 15.2% with a median disease-free survival of 5.2 months (69). In 1996, a landmark study by Mazzaferro et al. defined selection criteria for HCC patients, the so-called Milan criteria (70). With the Milan criteria, a subgroup of HCC patients could be identified, consisting of patients with a single nodule up to 5 cm or <3 nodules <3 cm without extrahepatic manifestation and no vascular invasion, who achieve similar results after OLT as patients who receive OLT in end-stage-cirrhosis without HCC. Many have since adopted the Milan criteria and confirmed its success with a 5-year-survival of >70% (71, 72). In contrast, with increasing experience, multiple authors addressed the Milan criteria as being too restrictive. Careful selection of patients remains a matter of debate, the fact of the matter being the limited availability of liver donors worldwide (71–73).

Once eligible for OLT, patients are placed on a waiting list. Availability ranges from days to months. The incidence of disease progression while listed is 10–23% and death during evaluation is around 11% (24, 25). Since liver donors are scarce, bridging the period of listing is essential. This clinical setting is called “bridge to transplantation.” Many of the aforementioned modalities may be used in this particular setting. Regional control as a bridge to transplant by using either RE, TACE, RFA, resection, chemotherapy, or a combination of these modalities is usually safe, without affecting post-transplant survival (16). Both RE and TACE show promising results in gaining regional control. As a bridge to transplantation, time and quality of life play an important role.

Lewandowski et al. compared RE (*n* = 43) with TACE (*n* = 43) as a bridge to transplantation (11). In their study, the median time to progression was defined as the interval between PD by WHO response criteria and the time of treatment. The median time to progression for TACE vs. RE was 19.6 vs. 48.6 months, respectively (*p* = 0.008). According to the EASL criteria, the 1-year progression rate was 40% for TACE vs. only 8% for RE (*p* = 0.01). Given its durable response in HCC, RE might, therefore, be the preferred choice as a bridge to transplantation. Moreover, the group treated with TACE was hospitalized for 3 days on average and received a median of two treatments per patient. In contrast, the RE group was treated on an outpatient basis and received a median of one treatment per patient (11). As quality of life plays an increasingly important role in medical decision-making, these logistical benefits definitively favor RE over TACE in this setting. In the developing field of RE even single session outpatient procedures have been described, in which all procedures take 1 day in total (74). Additionally, after treatment with ≤3 GBq ^90^Y-microspheres no contact restrictions are necessary for patients and their families (75). Altogether these results are very promising, but need to be reproduced in larger patient populations including quality of life investigations.

Transplantation may not be restricted to HCC alone. Other primary liver malignancies have shown promising results as well, like CCs and hepatic epitheloid hemangioendotheliomas (76).

When it comes to secondary malignancies that are limited to the liver, well-differentiated NET have been investigated for OLT too. Primary treatment of a NET includes resection of the primary tumor. Dissemination usually occurs at a later stage and is often limited to the liver only. In NET, 60–70% of patients present with diffuse, multifocal liver metastases, ineligible for radical treatments (77). When NET patients present with limited liver metastases, surgical resection results in only 10–25% curative resections with a 5-year recurrence rate of around 80% (78).

Orthotopic liver transplantation may provide the best curative treatment option for patients with metastatic NET, similar as to OLT in HCC. Both producing NET and non-producing NET are eligible for OLT and selection criteria for NET include the Milan criteria of 2007 (78), which are adopted by the European Neuroendocrine Tumor Society guideline of 2012 (77). Logically, the Milan Criteria for NET are different from the Milan criteria for HCC. They include only histologic confirmed well-differentiated tumors, liver tumor load <50%, age <50 years and stable disease for at least 6 months prior to OLT. With a 5-year-survival up to 90% (range 33–90%) OLT seems promising in NET patients matching these criteria. However, tumor recurrence after transplantation may eventually pose a problem with a 5-year disease-free survival rate ranging from 20 to 77% (79).

Radioembolization as a bridge to transplantation in NET may have some benefit. As mono-treatment, the largest study to date on RE in NET patients (*n* = 148) reported a response rate of 63% and a disease control rate (defined as CR + PR + stable disease) of 86%, combined with a median survival of 70 months (56). With such efficacy, RE may provide effective bridging in NET patients. No studies have been performed to investigate this hypothesis. Currently in other secondary liver malignancies, OLT has no place in the treatment algorithm. Some have used OLT in CRC, but within 2 years essentially all patients developed disease recurrence (76).

## Future Remnant Liver

As surgical techniques evolve, more patients will be candidates for extensive liver surgery. Resections of liver segments or complete lobes are well tolerated. However, careful patient selection is crucial to avoid liver failure due to limited hepatic reserve after resection. According to current standards, the FRL should account for more than 25% of the total liver volume (TLV). In patients with underlying chronic liver diseases (like cirrhosis) this should be more than 40% of TLV (80). Both cut-off values are based on volumetric measurements on radiologic imaging, computed tomography, or magnetic resonance imaging (CT or MRI).

Once patients are screened for resection and FRL is deemed inadequate, PVE of the tumor-bearing lobe is often considered to gain hypertrophy of the FRL, the non-embolized lobe. After PVE, adequate hypertrophy can be accomplished in 3–6 weeks, and extensive resections can be permitted (80). In cirrhotic livers and patients formerly treated with chemotherapy (especially platinum compounds), hypertrophy of the FRL after PVE may be insufficient (81, 82). After PVE, hypertrophy of the FRL may range from 8.5% up to 69% (82). Several clinics have noticed a similar phenomenon of hypertrophy of the non-treated lobe after RE (83–88). Table 4 summarizes findings of mainly retrospective studies on the degree of hypertrophy (DoH) of the non-treated lobe. DoH is defined as the FRL volume minus the FRL volume before treatment, divided by the FRL volume before treatment. A DoH of approximately 35% has been observed at 3–4 months after RE (range 8.9–57%). Garlipp et al. compared RE (*n* = 35) with PVE (*n* = 141). In their population, PVE resulted in significantly more hypertrophy of the non-treated lobe compared to RE after 1 month (61.5 vs. 29%) (88). The main limitation of this study was the follow-up interval of 1 month. This might have been too short to observe sufficient hypertrophy.

**Table 4:** **Hypertrophy after RE**.

Reference	Patients	Follow-up	Volume	Degree of hypertrophy	Degree of atrophy
		period	measurement	contralateral lobe (%)	treated lobe (%)
Jakobs et al. (83)	32	139 days	CT/MRI	8.9	21.2
Gaba et al. (84)	20	3 months	CT/MRI	40	52
Ahmadzadehfar et al. (85)	24	44–66 days	MRI	57	6
Edeline et al. (86)	34	3 months	CT	29	23
Vouche et al. (87)	83	1 month	CT/MRI	7	2
		3–6 months		35	21
		>9 months		45	32
Garlipp et al. (88)^a^	35	46 days	MRI	29	NA
	141^†^	33 days^†^		61.5^†^

*NA, data not available*.

*^a^Only prospective study*.

*^†^RE vs. PVE, PVE results are marked*.

We have learned from studies with living donor LTx that the liver has a steady pace in regeneration. In a study by Klink et al., after donation of a right liver lobe, the remaining left lobe had a mean volume of 36.1% of the TLV (baseline) (89). After 1 month, FRL was 54.8% of the pre-transplantation TLV (53.6% DoH). After 3 months, 80% of the pre-transplantation TLV was restored (146% DoH) and after 12 months the post-transplantation volume was equal to or even more than 100% of the initial TLV (267% DoH) (89). This shows the value of a longer interval between the induction of hypertrophy and surgery.

Vouche et al. showed a similar dynamic pattern in RE (Table 4) with a DoH of 7% at 1 month, 35% at 3 months, and 45% at 9 months (87). In comparison, Corrêa et al. showed that PVE resulted in a 50% DoH occurring in the first 90 days (approximately 3 months) and 75% by 230 days (approximately 8 months) in patients who were not eligible for resection after PVE. The study by Corrêa et al. included patients who experienced PD during the time interval between PVE and surgical resection. This corresponded with 26% of the total population treated with PVE (90).

Resection should be performed shortly after PVE, since significant tumor progression may be seen in the PVE lobe and tumor progression can affect the FRL. Comparable to the previously mentioned study by Corrêa et al. and other studies, a study by de Baere et al. showed a high rate of patients with tumor progression after PVE, rendering them ineligible for resection (81, 90). De Baere et al. treated 106 patients with PVE and showed a DoH of the FRL of 69% obtained within 27–52 days (mean 31 ± 5 days). Subsequently, successful hemi-hepatectomies were performed in 88%, but 12% were deemed inoperable due to tumor progression, extrahepatic spread, or liver metastasis in the hypertrophic lobe (81). In a recent analysis by Vyas et al., 17.5% of the patients experienced tumor progression and 4.8% had failure of hypertrophy prior to surgery in a pooled population of 1532 patients undergoing PVE (26).

Although at a slower rate, RE can induce substantial hypertrophy in the non-treated lobe while treating the tumor at the same time. This may lead to less tumor progression during the interval between RE and surgery. Unfortunately, no prospective trials have yet been performed to investigate this hypothesis.

Another way to prevent tumor progression is to simply perform surgery sooner, before the tumor has the chance to progress. There are some studies suggest that FLR volume is a suboptimal predictor of post-operative liver failure. Ideally, we should look at FRL *function* to predict the chance of success (91, 92). This especially becomes relevant in patients with underlying liver disease, in whom some parts of the liver might have a better function than other parts. There are different methods for assessing liver function. First assessment of liver function is usually done by measurements of liver enzymes (aminotransferase levels and alkaline phosphatase) and products indicative of liver synthesis such as albumin, bilirubin, and prothrombin time in blood. However, liver enzymes are markers of liver injury and products of hepatic synthesis function can be affected by different extrahepatic factors such as nutrition, hemolysis, antibiotic use, and systemic illness.

The most well-known and applied dynamic quantitative liver function tests are the indocyanine green clearance (ICG) and galactose elimination capacity. ICG is a tricarbocyanine dye, cleared from the plasma by hepatocytes and excreted into the bile. The ICG clearance test is considered the most accurate test to evaluate the hepatic functional reserve before surgery and to predict post-operative mortality (93). The carbohydrate galactose is metabolized nearly exclusively in the liver. The elimination rate of galactose from the blood depends on the phosphorylation of galactose by galactokinase. Both these dynamic tests, in which multiple blood samples need to be taken, have been shown to predict post-operative complications and mortality (94, 95). However, they are not able to tell the surgeon how much of the liver can be resected safely or whether there are regional differences in liver function.

In order to appreciate regional differences in liver function, you have to make them visible. In the past years, two different nuclear medicine imaging techniques for assessment of liver function have been developed: ^99m^Tc-galactosyl human serum albumin (^99m^Tc-GSA) and ^99m^Tc-IDA. ^99m^Tc-GSA scintigraphy measures the binding of ^99m^Tc-GSA to its receptor (the asialoglycoprotein receptor), which is expressed on functional hepatocytes only. Liver function measured by ^99m^Tc-GSA scintigraphy correlates well with conventional liver function parameters, including the ICG clearance test (96, 97). ^99m^Tc-GSA has been shown to be of value for pre-operative risk assessment of partial hepatectomy (97, 98). One major limitation is the availability of ^99m^Tc-GSA, as it is only available for clinical use in Japan.

**Figure 1:**
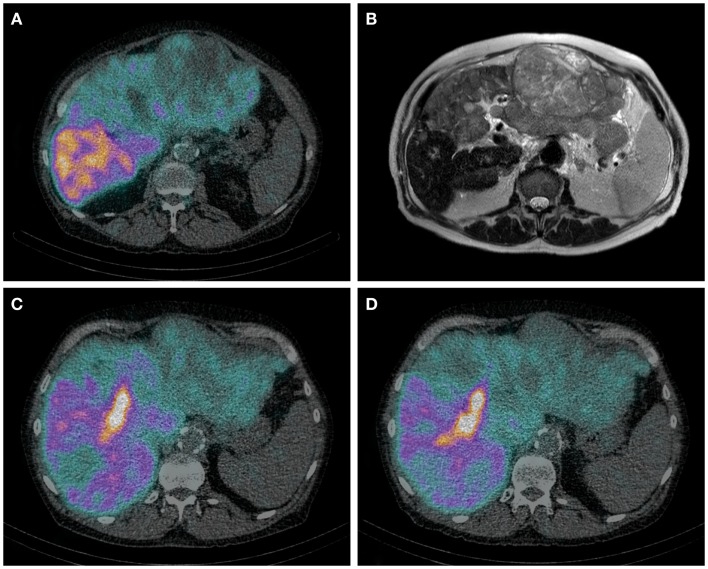
**(A–D)** Axial HBS SPECT-CT image through the abdomen of a patient with hemochromatosis and multifocal HCC. Notice the regional uptake differences in cirrhotic and tumorous tissue **(A)**. Corresponding MRI T2 weighted image **(B)**. Same patient before **(C)** and after **(D)** RE of the left lobe including segment 4. **(C)** It is from the same HBS SPECT/CT as image **(A)**, but shown in a different axial plane. The decrease of ^99m^Tc-mebrofenin uptake after treatment is best visible in segment 4. The area of high uptake is biliary excretion in a dilated bile duct.

Hepatobiliary scintigraphy using ^99m^Tc-iminodiacetic acid analogs (^99m^Tc-IDA), has been used since the 1970s for the scintigraphic evaluation various biliary diseases. After uptake by organic anion transporter peptides expressed on the hepatocytes, IDA analogs are excreted in the bile by ATP-dependent export pumps, without undergoing biotransformation (99). Therefore, IDA agents are ideal tracers for the biliary tract. More recent, HBS with IDA analogs has been used to evaluate liver function. Liver uptake of IDA analogs can be influenced by high plasma levels of bilirubin (100). Of all IDA analogs, ^99m^Tc-mebrofenin has the strongest resistance to displacement by high bilirubin concentrations and it also has the highest hepatic extraction fraction. For these reasons, ^99m^Tc-mebrofenin is the most favorable IDA analog. Erdogan et al. have shown that the hepatic uptake rate of ^99m^Tc-mebrofenin correlates well with the ICG clearance rate and is an efficient method for determining liver function (101). The same group from Amsterdam later showed that pre-operative HBS is more valuable in estimating the risk of post-operative liver failure than CT volumetry in patients with underlying liver disease (102). They provided a FRL function cut-off value for the prediction of post-operative liver failure. Because HBS is a pure functional test, this cut-of-value is the same for patients with or without underlying liver disease. Therefore, it can be used in patients with a pre-operative unknown quality of liver parenchyma (99) (Figures 1A–D).

With the use of SPECT-CT and CECT, assessment of liver function at a segmental level becomes possible. Combining the functional data from the SPECT and the anatomical information of the CECT will enable an even more accurate estimation of the post-operative liver function in the future.

There have been few studies on the effect of PVE on liver function compared to liver volume. In a study of 24 patients by De Graaf et al., FRL volume and function were assessed 3–4 weeks after PVE. FRL function increased significantly more than FRL volume (103). Using ^99m^Tc-GSA, other studies also describe that the increase in function is more pronounced than the increase in volume (91, 92). These findings suggest that the time between PVE and surgery may be shortened, thereby leaving less time for tumor progression.

## Discussion

In the previous sections, we described RE as an interval treatment modality for downstaging, bridge-to-transplant and future remnant hypertrophy. Although level 1 evidence is lacking, preliminary results show promising accuracy of RE for these particular indications.

Downstaging is feasible in patients with HCC as shown by several authors (11, 49–53), but it should not be limited to HCC alone, since response rates of other liver malignancies by RE are similar or even higher, including a long-lasting effect. As described in our review, gaining surgical eligibility leads to survival prolongation. If patients are treated with an intention to downstage and when sufficient tumor reduction has occurred, selected cases might even be eligible for OLT, which is the most promising curative treatment for several primary and secondary liver malignancies at this time, as discussed in our review. If patients are not eligible for OLT and the future liver remnant is deemed insufficient, the later making the patient ineligible for resection as well, inducing hypertrophy is paramount. In achieving sufficient hypertrophy RE might be preferred over PVE, because RE has the advantage of the combination of hypertrophy induction and local disease control. Success of a combination of simultaneously downstaging and inducing hypertrophy has already been described in a case report by Gulec et al. (104).

Use of RE for downstaging, bridge-to-transplant and attaining an adequate FRL seems very promising; however, several related procedures need refinement too. Current imaging modalities and their response criteria are incapable of predicting the degree of tumor necrosis in lesions treated by RE (49, 53). A better determination of the degree of necrosis could assist clinicians in personalizing treatment algorithms and might even define the timing of applying treatment (alternations). In improving related imaging for these indications, hepatobiliary scintigraphy is taking the lead. By determining liver function instead of liver volume, eligibility for surgical resection could be attained sooner by evaluating function gain in the FLR, since functional and volume gain do not go hand in hand. Furthermore, hepatobiliary scintigraphy takes an underlying liver disease into account, like cirrhosis, which is not uncommon in patients with HCC.

At the same time, dosimetry is crucial to optimize RE in these settings. Theoretically, the higher the tumor absorbed dose, the more effective. This rationale was supported by (pre-)clinical studies in different settings (105–108). However, although the surrounding normal liver cells are affected less, high activity levels can result in loss of healthy liver parenchyma. Thus, the goal is to find the right balance between maximum tumor absorbed dose and preservation of healthy tissue in each individual patient. As briefly pointed out in our introduction on RE, multiple activity calculation methods are being used. When using resin microspheres three activity calculation methods have been described (Table 1). The empirical method that was solely based on tumor load has been abandoned due to an unacceptable toxicity profile and the lack of any patient-individualized factors (29, 109). The BSA-method (semi-empirical) has been used safely in many clinical trials and is recommended in patients with concurrent or previous chemotherapy by the manufacturer (30). It is easy to use in daily practice and has strong historical data (77). However, this method has been criticized in literature in many aspects, mostly based on not taking liver volume into consideration. As a result, under- (small patient + large liver) or overtreatment (large patient + small liver) can occur (109, 110). Additionally, the BSA-method does not take the tumor-to-non-tumor ratio (T/N ratio) into account, which is to the disadvantage of patients with hypervascular tumors who could withstand higher administered activities. The third calculation method, the partition model, embeds many of these relevant factors. It takes into account the T/N ratio, tumor volume, and liver volume. All variables in this equation can be acquired from the ^99m^Tc-MAA-SPECT/CT prior to RE, so no additional procedures are needed (111). Only poorly delineated tumors pose a problem for quantification. The complexity of the partition method makes its use less attractive in daily practice. In daily practice, the BSA-method is most commonly applied method for dose calculation (111). Nonetheless, the partition model based on the ^99m^Tc-MAA-SPECT/CT findings should be preferred by clinicians (111).

In the case of glass microspheres, the manufacturer advocates one activity calculation method (Table 1), in which the T/N ratio has not been included (31). Like the discussion surrounding activity calculation for resin microspheres, treatment based on prior ^99m^Tc-MAA-SPECT/CT has been shown feasible for glass microspheres and seems very promising (108). One should bear in mind though that the result of the ^99m^Tc-MAA-SPECT/CT is influenced by many factors, causing discrepancies between ^99m^Tc-MAA-particles distribution and ^90^Y-microspheres distribution on which dose calculation is based, when applying the partition method (summarized in Table 5).

**Table 5:** **Factors causing ^99m^Tc-macroaggregated albumin (^99m^Tc-MAA) and ^90^Y-Microspheres (^90^Y-MS) distribution discrepancy (27, 110, 112, 113)**.

**Procedural aspects**
Catheter positioning	Similar positioning in both angiographies
	Equal proximity to bifurcations
Injection rate	Bolus or rapid (MAA) vs. intermitted delivery (^90^Y-MS)
**Particle aspects**
Particle flow dynamics	Randomly formed ^99m^Tc-MAA vs. spherical ^90^Y-MS
Administered amount	^99m^Tc-MAA ± 150.000 particles vs. ^90^Y-MS ±4–50 million particles
**Technical aspect**
Patient positioning	Registration mismatch between scans
Shortcomings imaging	Variability in delineation of tumors
	Threshold definition of tumor vs. non-tumor
Scanning modality	^90^Y-Brehmstralungs-SPECT/CT vs. ^90^Y-PET/CT
Breathing artifacts	Registration difficulties between scans
**Patient factors**
Primary tumor	Ability of tumor delineation on imaging
Vascular	Artery spasms during delivery
	Stasis of flow during ^90^Y-MS administration

As mentioned in our short introduction to RE, ^90^Y-brehmsstralung SPECT/CT or ^90^Y-PET/CT is acquired post-treatment to evaluate the distribution of the microspheres. At the same time, both modalities can be used for post-treatment dosimetry. Currently, ^90^Y-PET/CT is favored over ^90^Y-brehmsstralung SPECT/CT by many RE-centers (114–119). Calculating the administered tumor absorbed dose on post-treatment imaging gives insight into the expected response. Several studies showed that the tumor absorbed dose was correlated to the objective response (115, 117, 120, 121). Additionally, heterogeneity of the absorbed dose within the tumor can be assessed, which correlates with the partial/regional tumor response (115, 119, 120).

In downstaging and bridge-to-transplant, dosimetry should optimize the tumor absorbed dose, while delivering an acceptable to minimal dose on healthy liver tissue. Applying the partition method based on the ^99m^Tc-MAA-SPECT/CT is preferable and when possible, super-selective catheterization of the tumor-bearing lobe can be considered to further improve tumor dose and healthy liver dose differences. By doing so, minimizing radiation induced complications and preserving healthy liver tissue, which is badly needed after surgical resection. However, the vast majority of the current studies did not use dose calculations based on the ^99m^Tc-MAA-SPECT/CT, so optimal dose calculation might not have been reached and downstaging success rates could be even higher (11, 49–53). Additionally, minimizing lung shunting and preserving lung function before an intensive liver transplant procedure or large surgical resection, may become an additional aspect to consider. In LTx, peri-operative death occurs in 5.3–7.0%, mostly due to multiple organ failure (including respiratory insufficiency) and approximately 42.1% of all patients develop pulmonary complications (pneumonia and pleural effusion) after LTx (122, 123). In current studies performing RE as a bridge to transplantation, none specifically mentioned pulmonary complications after OLT (11, 49–53).

In the induction of FRL hypertrophy, the underlying mechanism of liver hypertrophy remains a mystery (82). Since the embolic effect of RE is less substantial than in PVE, remnant hypertrophy after RE might largely be based on an irradiation induced effect in the treated liver lobe. This causes fibrosis, leading to increased portal pressure and eventually to shunting of portal venous blood away from the irradiated fibrotic lobe to the untreated contralateral lobe by preferential flow (83, 84, 86). This effect and its results do not arise as rapidly as in PVE, as described by Vouche et al. and Corrêa et al. (87, 90). After PVE, a more macroscopic occlusion creates a sudden shunt of portal venous blood to the untreated lobe. In some cases, repeated RE resulting in a higher cumulative dose led to an increase in hypertrophy of the untreated lobe (50). Only Edeline et al. found no correlation between the absorbed dose and hypertrophy in their study (86). That study was soon followed by a multivariate analysis of Vouche et al., in which the absorbed dose was no significant variable (87). Nonetheless, no studies have been performed solely to investigate this phenomenon and its relation to dose.

Apart from assessing FRL function with HBS, HIDA could be a very interesting modality when it comes to RE. At present, there have been no studies evaluating the effect of RE therapy on liver function apart from laboratory toxicity. In the future, scintigraphy can be used to learn us more about changes in liver function after RE, for example in relationship to microsphere distribution and dose. Another area of research could be time to functional recovery after RE, which in turn could potentially be helpful in determining when to perform repeat RE treatment if needed.

## Conclusion

Trans-arterial treatment of liver malignancies with RE is an emerging treatment modality. RE is predominantly performed in patients with no curative options, mostly in a salvage setting. Potentially curative settings in which RE may be applied include downstaging patients to resec disease, a bridge to transplantation and induction of remnant liver hypertrophy. RE involves a combination of tumor reduction and disease control, minimizing the chance of tumor progression during the time interval prior to liver surgery with curative intent. This may eventually lead to prolonged survival, although prospective controlled trials are needed to test this hypothesis. Imaging is indispensable for patient selection and dosimetry-based treatment planning to use the full potential that RE has to offer in patients with liver malignancy, especially when liver surgery with curative intent might still be an option.

## Conflict of Interest Statement

Solely the publication costs are kindly funded by SIRTeX Medical. No other conflict of interest.
